# The Decline of Tuberculosis

**Published:** 1899-10-07

**Authors:** 


### THE DECLINE OF TUBERCULOSIS.

In a paper read before the recent Health Congress at Blackpool, Dr. Ransome, F.R.S., showed that the mortality from phthisis in England and Wales had diminished by one-half in the course of the last 35 years, and, although we could not entirely trust the earlier returns of the Registrar-General, it was extremely probable that it had really declined to one-third of its prevalence 60 years ago. As to the cause of this enormous shrinkage he could but attribute it to the better drainage of the soil, to improved dwelling accommodation, and to a greater power of resistance on the part of the population against infection, and he thought that in the future, as in the past, we should have to rely mainly on the public health service for thorough-going measures against tuberculosis. After describing the various proceedings which might be adopted by the local sanitary authorities, he again reverted to the point that sanitation in the true sense of the word extended beyond the special sphere of work commonly delegated to sanitary departments, and included improvements in the condition of the working population, in their feeding and clothing, and especially in their housing, in their hours of labour, and in the protection given them from noxious atmospheres. These were matters which needed the co-operation of other than merely sanitary bodies, and a strong and wholesome public opinion on the subject must be cultivated before much good could be done. As to the rôle of the microbe, Dr. Cartwright, of King's College, thought it was a question whether ordinary sanitation could reduce the prevalence of tuberculous disease beyond a certain point. We had yet to find out what it was that determined whether the disease would get a foothold in those who were exposed to it, for myriads of germs passed through the system for every one that succeeded in getting an entrance into the tissues, and until we know more about that aspect of the question our precautionary measures were at the best empirical. This is good teaching. Above all things, it is important that the truth should be insisted on that, in the propagation of tuberculosis, there is something far more important than the microbe.

